# Changes in pupil dilation and P300 amplitude indicate the possible involvement of the locus coeruleus-norepinephrine (LC-NE) system in psychological flow

**DOI:** 10.1038/s41598-023-28781-z

**Published:** 2023-02-02

**Authors:** Hairong Lu, Dimitri van der Linden, Arnold B. Bakker

**Affiliations:** 1 Department of Psychology, Education, and Child Studies, Erasmus University Rotterdam, 3062 PA Rotterdam, The Netherlands; 2 Department of Industrial Psychology and People Management, University of Johannesburg, Johannesburg, South Africa

**Keywords:** Psychology, Human behaviour

## Abstract

Psychological flow is a state of full task immersion. The present study was conducted to test the hypothesis that psychological flow is positively related to activity of the phasic locus coeruleus-norepinephrine (LC-NE) system, which supports decisions on whether to engage in or disengage from the current activity. Subjective flow was assessed among 36 participants who engaged in a gamified version of the *n*-back task with various difficulty levels (0, 1, 2, and 3 back). During the tasks, continuous pupil diameter and EEG were recorded. We found that psychological flow and two presumed indicators of the phasic LC-NE activity (pupil dilation and EEG P300 amplitude) fit inverted U-shapes with increasing subjective task difficulty. Moreover, a positive linear relationship between psychological flow and pupil dilation (not with P300) was found. In conclusion, this study indicates the involvement of the LC-NE system in the peak experience of flow.

## Introduction

Exerting mental or physical effort is often considered aversive as it involves psychological and physiological costs^1–3^. Yet, it is also well-established that in real life, people frequently engage in and invest great effort into tasks they interested in^4,5^. One line of research that focuses on this latter phenomenon relates to psychological flow^6^, henceforward ‘flow’. Flow is a subjective experience generated from intense engagement in an activity, up to the point where one seems to forget the surrounding and has a distorted time perception (time seems to fly). Mihaly Csikszentmihalyi, who coined the term in the Seventies, interviewed people in various disciplines and found that they describe their optimal task experience in very similar ways. That is, they often refer to it as being in the ‘flow’ of things^7^. As such, flow refers to a mental state of full task immersion. While numerous studies have now shown that flow is relevant for optimal performance and well-being in various life domains^8–11^ relatively little is known about its underlying neural mechanisms^12–14^. This is unfortunate, because knowing the neurocognitive underpinnings of the state may help to better understand the nature of flow.

In line with this, Van der Linden et al.^13^ recently proposed a neuropsychological model of flow. One of the neural structures they hypothesized to be involved is the locus coeruleus-norepinephrine (LC-NE) system. The locus coeruleus is a small nucleus located deep in the pons and it sends dense efferent projections to almost all brain regions modulating large scale neuronal activity. It is the only source of the norepinephrine to cerebral, cerebellar, and hippocampal cortices. Van der Linden et al.^13,15^ argued that the psychological processes that have been associated with LC-NE activity, such as optimal task engagement^16,17^, show considerable overlap with the characteristics of flow. Moreover, in flow as well as LC-NE research, it is assumed that task engagement associated with an inverted-U shape of arousal^16,18^. Accordingly, in the present study, we combine subjective and physiological measures of task engagement in order to test whether presumed indicators of the LC-NE system relate to flow.

In their seminal papers, Aston-Jones and Cohen^16,17^ argued that the LC-NE system has two different activity modes, namely phasic and tonic. Tonic LC-NE activity refers to the baseline NE release, while phasic LC-NE activity is characterized by quick bursts of NE release as a response to stimuli. They suggested that the decision to put further effort into a task rather than searching for more rewarding activities associates with the strong phasic and intermediate tonic modes of the LC-NE. Specifically, optimal engagement and effort put into the task at hand is accompanied with a moderate tonic NE output, combined with relatively strong stimulus-coupled phasic NE responses^19^. This basically implies that during this LC-NE ‘high-engagement mode’ (i.e., moderate tonic, strong phasic activity), a person is likely to be highly immersed in a task and shows optimal performance.

It was noted that these characteristics of the LC-NE system in relation to task performance show remarkable resemblance to key characteristics of the psychological flow state^15^. Flow is indicated by full task immersion, optimal performance^8,20,21^, and intermediate arousal^18^. Hence, we hypothesize that, to some extent, flow may be a psychological manifestation of LC-NE activity being in the peak of the engagement mode. Although there are various possible approaches (e.g., fMRI) to test the relationship between LC-NE activity and flow, in this study, we adopt a psychophysiological approach that uses dynamic and low-cost presumed indicators of the LC-NE system: pupil diameter and the well-known P300 event-related potential in electroencephalographical (EEG) measures.

Human and animal experiments revealed that LC-NE activity closely matches moment-to-moment changes in pupil size^22–24^. In a range of experiments using human participants, researchers confirmed that pupil dynamics mimic the various modes of the LC-NE system during task performance^25,26^. For example, these scholars found that when engaging in a task that was deemed rewarding, participants showed moderate levels of baseline (tonic) pupil size, combined with strong stimulus-related pupil responses. One relevant aspect of these pupillometric studies is that it became clear that the type of mode participants were in was highly dependent on the match between the participants’ skills and the level of task challenge. To illustrate, Gilzenrat et al.^25^ designed an experiment in which participants performed a discrimination task that gradually went from easy trials with low incentives, to difficult trials with higher incentives. The researchers found that when participants considered the trials too difficult to obtain sufficient rewards, they made use of the possibility to reset and start with the easy-low reward trials again. Interestingly, the decision to ‘reset’ was preceded by a pattern of increasing baseline pupil dilation accompanied with diminished stimulus-evoked pupil responses. In other words, pupil dynamics tracked whether the participants considered their skills are adequate to deal with the task at hand. Gilzenrat et al.^25^ interpreted these findings as support for the notion that the LC-NE system plays a role in task engagement. Accordingly, we argue that the phasic pupil response (strong stimulus-evoked pupil dilation combined with a moderate baseline pupil size) is related to the skill-challenge balanced psychological flow state.

Another psychophysiological indicator we use in this study, the P300, has long been known to be regulated by attention and task demands^27,28^. Studies suggest that the P300 may represent a cortical electrophysiological correlate of phasic LC responses^29–31^. This proposition is in line with earlier animal studies. For example, Swick and colleagues^32^ found that systemic clonidine, an alpha-2 adrenergic agonist medication, increased the latency of a P300-like potential in monkeys. More recently, Murphy et al.^30^ conducted an experiment with human participants using an auditory oddball task. They found a quadratic relationship between baseline pupil diameter and P300 amplitude that closely mirrored the changes in task engagement as predicted by theories on the role of LC-NE functions in performance. Moreover, it seems that task difficulty induced P300 amplitude follows this inverted U-shape as well^33,34^ For example, in a series of mathematical tasks that gradually increased in difficultly level, the P300 amplitude showed an inverted U-shape from 'extremely easy' to 'extremely difficult' task^33^. Using a dual task, Nune et al.^34^ indeed found that P300 amplitude elicited by the secondary task was reduced in a flow condition compared with boredom and overload conditions, indicating that more attentional resources were allocated to the main task during flow. Even though some have suggested that the P300 amplitude mainly decreases progressively as task difficulty increases^35,36^, it is still reasonable to argue that the real effect of task difficulty may be cloaked by the limited range of task difficulty. For example, a recent study suggested that higher levels of task-induced boredom were associated with reduced amplitudes for P300^37^. This implies that individual perception of the task, rather than objective task difficulty influenced the P300. Therefore, we argue that the LC-NE activity indexed by P300 amplitude is inverted U-shaped related to task difficulty, especially subjective task difficulty.

In this article, we present an experiment in which we examined the relationships between psychological flow, pupil diameter, and the P300 amplitude. The selection of the psychophysiological indicators is based on LC-NE theory. We used a gamified version of the well-known *n*-back task with different difficulty levels to test whether (1) psychological flow, task-evoked pupil dilation, and the P300 amplitude all show the expected inverted U-shapes in relation to task difficulty; (2) there are positive correlations of task-evoked pupil dilation and P300 with psychological flow.

## Results

### Psychological flow in relation to objective and subjective task difficulty

**Figure 1:**
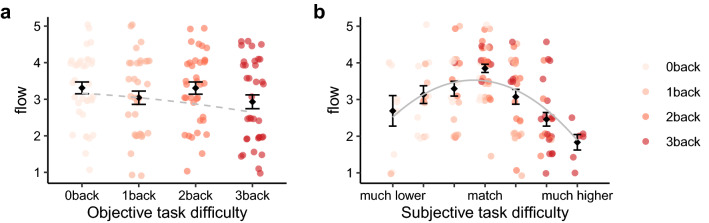
Psychological flow changes as a function of task difficulty. (**a**) Psychological flow failed to fit an inverted U-shape pattern with increasing objective task difficulty. (**b**) Psychological flow fit an inverted U-shape pattern with increasing subjective task difficulty. The means ± standard errors are shown.

First, we tested whether the task difficulty (objective and subjective task difficulty) related to psychological flow in an inverted U-shape. We did this by adding a quadratic item into the linear mixed model^38^. Results showed that the relationship between objective task difficulty and psychological flow (Fig. 1a) did not fit a quadratic model (objective task difficulty: *β*_(*OTD*_^2^_)_ = − 0.026, *p* = 0.714, B = 0.032, Conditional R^2^ = 0.341, Marginal R^2^ = 0.009, *Δχ*^2^_(1)_ = 0.137, *p* = 0.711). However, the relation between subjective task difficulty and flow (Fig. 1b) followed a quadratic trend and the quadratic model fit significantly better to the data than the linear model (subjective task difficulty: *β*_(*STD*_^2^_)_ = − 0.145, *p* < 0.001, B = − 0.330, Conditional R^2^ = 0.401, Marginal R^2^ = 0.171, *Δχ*^2^_(1)_ = 29.482, *p* < 0.001). Detailed model comparisons are listed in Supplementary Table S2. This later finding is in line with flow theory and the mainstream of flow studies^8,9,39,40^, suggesting that the highest flow experience appears when individuals perceived a match between their skills and task difficulty.

### Pupil diameter in relation to objective and subjective task difficulty

**Figure 2:**
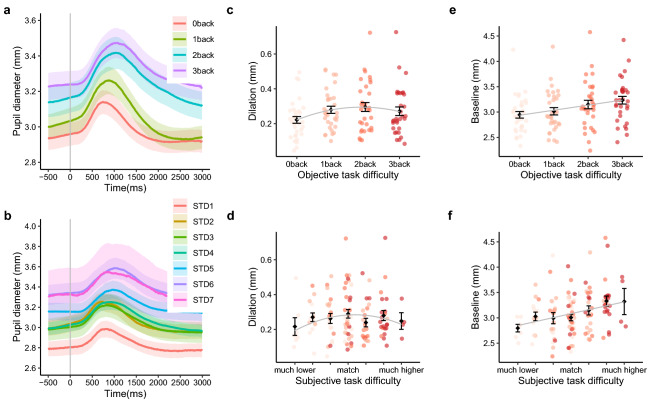
Pupil diameter changes as function of task difficulty. (**a**) Grand averaged continuous pupil diameter in four n-back tasks. (**b**) Grand averaged continuous pupil diameter in seven subjective task difficulty groups (*STD* subjective task difficulty). (**c**) Quadratic relationship between objective task difficulty and stimuli-evoked pupil dilation. (**d**) Quadratic relationship between subjective task difficulty and stimuli-evoked pupil dilation. (**e**) Linear relationship between objective task difficulty and baseline pupil diameter. (**f**) Linear relationship between subjective task difficulty and baseline pupil diameter. The means ± standard errors are shown.

Averaged continuous pupil diameter changes across time in four *n*-back tasks are shown in Fig. 2a. Averaged continuous pupil diameter changes across time in seven subjective task difficulty groups are shown in Fig. 2b. The pupil dilated after the stimuli appeared on the screen. The peak of the stimuli-evoked pupil dilation was located in the time window between 500 and 2000 ms after the stimuli.

Subsequent analysis revealed that the pattern of stimuli-evoked pupil dilation over increasing objective task difficulty (Fig. 2c) and subjective task difficulty (Fig. 2d) fit quadratic models (objective task difficulty: *β*_(*OTD*_^2^_)_ = − 0.021, *p* < 0.001, B = − 0.200, Conditional R^2^ = 0.778, Marginal R^2^ = 0.037; subjective task difficulty: *β*_(*STD*_^2^_)_ = − 0.008, *p* < 0.001, B = − 0.139, Conditional R^2^ = 0.719, Marginal R^2^ = 0.032). The significant negative quadratic coefficients in the tested models indicate inverted U-shape relationships. Further model comparison showed that the quadratic model fit significantly better to the data than the linear models for objective task difficulty and subjective task difficulty separately (objective task difficulty: *Δχ*^2^_(1)_ = 11.103, *p* < 0.001; subjective task difficulty: *Δχ*^2^_(1)_ = 8.758, *p* < 0.01). See detailed model comparisons in Supplementary Table S2.

In line with the LC-NE theory of task engagement, baseline pupil diameter showed a significant positive linear trend with increasing objective task difficulty (Fig. 2e) and subjective task difficulty (Fig. 2f, objective task difficulty: *β*_(*OTD*)_ = 0.100, *p* < 0.001, B = 0.259, Conditional R^2^ = 0.897, Marginal R^2^ = 0.066; subjective task difficulty: *β*_(*RTD*)_ = 0.078, *p* < 0.001, B = 0.272, Conditional R^2^ = 0.878, Marginal R^2^ = 0.074).

### EEG P300 in relation to objective and subjective task difficulty

**Figure 3:**
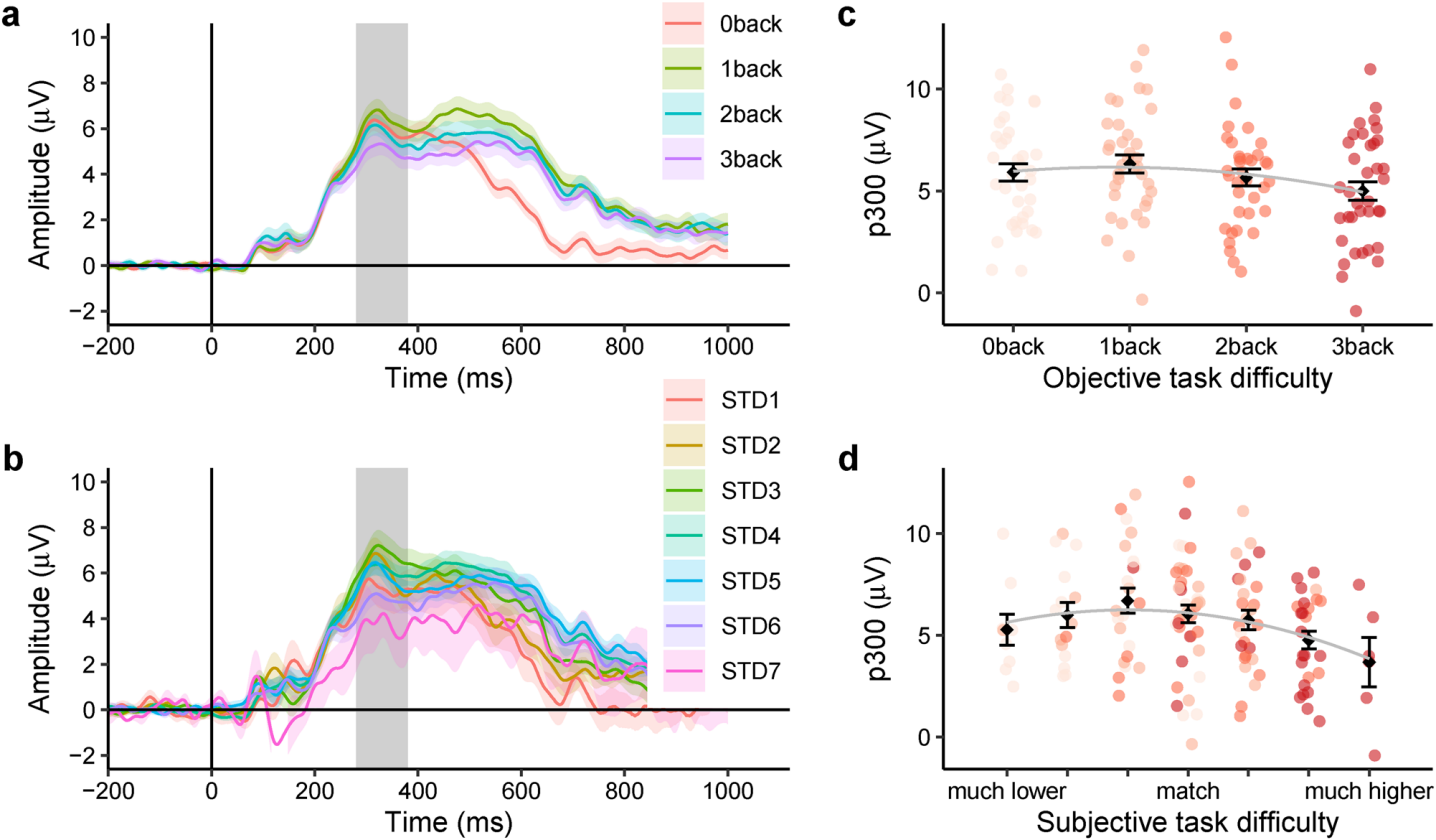
Stimulus-locked ERP changes in different task difficulty conditions. (**a**) Grand averaged continuous ERP waveform in four n-back tasks. (**b**) Grand averaged continuous ERP waveform in seven subjective task difficulty groups (*STD* subjective task difficulty). The grey shading indicates the time window where we extract the P300 amplitude value. (**c**) Quadratic relationship between objective task difficulty and EEG P300 amplitude. (**d**) Quadratic relationship between subjective task difficulty and EEG P300 amplitude. The means ± standard errors are shown.

Grand averaged continuous ERP waveforms of the Pz electrode in four *n*-back tasks are shown in Fig. 3a. Grand averaged continuous ERP waveform of the Pz electrode in seven subjective task difficulty groups are shown in Fig. 3b. It can be seen that the deflections were maximal between 280 and 380 ms.

The pattern of the P300 amplitude over increasing objective task difficulty (Fig. 3c) and subjective task difficulty (Fig. 3d) fit quadratic models (objective task difficulty: *β*_(*OTD*_^2^_)_ = − 0.270, *p* < 0.05, B = − 1.29, Conditional R^2^ = 0.741, Marginal R^2^ = 0.031; subjective task difficulty: *β*_(*STD*_^2^_)_ =  − 0.151, *p* < 0.001, B = − 0.137, Conditional R^2^ = 0.744, Marginal R^2^ = 0.051). The significant negative quadratic coefficients in the tested models indicate inverted U-shape patterns. In addition, model comparisons show that the quadratic models fit better to the data than linear models for both objective and subjective task difficulty (objective task difficulty: *Δχ*^2^_(1)_ = 5.698, *p* < 0.05; subjective task difficulty: *Δχ*^2^_(1)_ = 11.103, *p* < 0.001). See detailed information regarding model comparisons in Supplementary Table S2.

### Correlations of pupil dilation and P300 amplitude with flow

We tested the relationship between psychological flow and pupil dilation as well as psychological flow and EEG P300 amplitude, respectively, by fitting mixed effect models. It is worth noting that by fitting mixed effect models where subject number was entered as random intercept, we obtain information about how pupil dilation and P300 amplitude change with increasing levels of flow at the *within-person* level.

**Figure 4:**
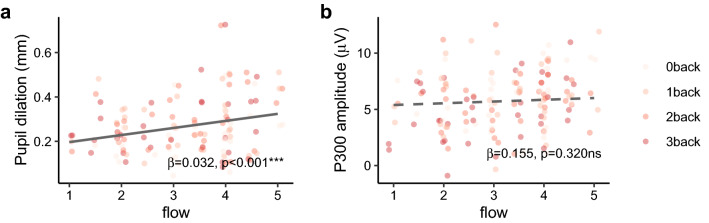
Correlations. (**a**) Linear relationship between pupil dilation and flow. (**b**) The relationship between P300 amplitude and flow failed to fit a linear model.

First, a significant positive linear relationship was found between pupil dilation and P300 amplitude (*β*_(*dilation*)_ = 4.178, *p* < 0.05, B = 0.204, Conditional R^2^ = 0.708, Marginal R^2^ = 0.041). When regressing pupil dilation on flow, we found a significant positive linear relationship (Fig. 4a, *β*_(*flow*)_ = 0.032, *p* < 0.001, B = 0.258, Conditional R^2^ = 0.718, Marginal R^2^ = 0.065). This means that when people reported more flow, their pupil also tended to dilate more toward the task stimuli. There was, however, no significant positive linear effect between flow and the P300 amplitude (Fig. 4b, *β*_(*flow*)_ = 0.155, *p* = 0.320, B = 0.062, Conditional R^2^ = 0.696, Marginal R^2^ = 0.004).

There is another explanation that the correlations of pupil dilation and P300 amplitude with flow might be due to the covariant effect caused by task difficulty. To rule out this possibility, we conducted regressions controlling for objective task difficulty. The result revealed that the effect of flow on pupil dilation remained significant even after controlling for objective task difficulty (*β*_(*flow*)_ = 0.0361, *p* < 0.001, B = 0.293, *β*_(*OTD*)_ = 0.020, *p* < 0.01, B = 0.173, Conditional R^2^ = 0.757, Marginal R^2^ = 0.100). This indicates that individuals’ flow experience explains unique variance in their increased pupil dilation. The effect of flow on P300 amplitude was non-significant when controlling for objective task difficulty (*β*_(*flow*)_ = 0.087, *p* = 0.562, B = 0.035, *β*_(*OTD*)_ =  − 0.333, *p* < 0.01, B = 0.142, Conditional R^2^ = 0.724, Marginal R^2^ = 0.022).

## Discussion

The present study examined a range of subjective and psychophysiological markers to test the possible involvement of the LC-NE system in regulating psychological flow. We hypothesized that (1) psychological flow and two phasic LC-NE activity indicators -task-evoked pupil dilation and the P300- all show inverted U-shapes in relation to task difficulty; (2) there are positive correlations of task-evoked pupil dilation and P300 with psychological flow. Our findings show that psychological flow, pupil dilation, and EEG P300 amplitude follow a similar inverted U-shape with increasing task difficulty. Moreover, we found that at the *within-person* level, individuals’ stimuli-evoked pupil dilation was positively related to their reported psychological flow, pointing to similar trajectories during the tasks. Based on the LC-NE theory of task engagement^16^, as well as the link between the LC-NE system and physiological indicators (cf.^25,26^), the current study provides preliminary support for the involvement of the LC-NE system in psychological flow.

Previous studies have shown that people engage more and get more pleasure from doing tasks that match their personal skill level^40–42^. Theoretical and empirical studies from neurophysiological field suggested that the LC-NE system plays a critical role in regulating the engagement–disengagement trade-off^16,29,43^. Combining findings from both psychological and neurophysiological literatures, the current study increases our understanding of the flow state in several ways. First, we showed that individual’s preference for tasks that match with their personal ability was reflected not only in psychological feelings, but also in physiological indicators. Specifically, similar inverted U-shaped patterns of pupil dilation and P300 indicate that individuals allocate more cognitive resources to matched tasks compared to too easy or too difficult tasks. Building on the assumptions that pupil dilation and P300 parallels with moment-to-moment changes of the phasic LC-NE activity, our findings that flow, pupil dilation and P300 amplitude all fit inverted U-shapes with increasing task difficulty provide support for the possible involvement of the LC-NE system in flow state.

Second, we showed that there is a positive within-person relationship between pupil dilation and flow. Specifically, we found that the pupil indeed dilates more during tasks in which individuals reported more flow. Previous studies suggested that pupil dilation reflects the moment-to-moment activity of the LC-NE system which represents the dynamics of task engagement^26^. Building on this notion, the present finding that the pupil dilates more when people report more flow, supports the hypothesis that flow is related to the phasic LC-NE regulated task engagement (cf.^13,15^).

Understanding the neurophysiological characteristics of flow is critical for understanding the nature of the mental state and for building a comprehensive theoretical framework of flow^12,14,15^. As reviewed recently by Alameda and colleagues^14^, we currently have a very limited scope in neurophysiological research in flow, which has hindered our understanding of its nature. The current study revealed the links between human pupil and brain responses during flow and draws our attention to the functions of the locus coeruleus. It provides useful insights and broads our scope in understanding the flow state.

In addition to the theoretical implications mentioned above, the present findings shed light on the implicit measurement of flow. Psychological flow has long been identified as a unique psychological phenomenon. However, its measurement on a momentary basis has always been problematic, since flow is a mental state that cannot be interrupted^44,45^. Any explicit measurement, like using statements to which participants need to respond, can only measure the mental state *after* the peak experience of flow. The eye is sometimes described as the window of an individual’s internal world^46^. Our study demonstrated that there is a positive association between pupil dilation and flow. This finding makes it possible to use pupil dilation as a non-invasive physiological indicator to capture the flow state implicitly.

While our findings were largely in line with our hypotheses and potentially provide new insights into the nature of flow, some findings remain open for interpretation. For example, the present study did not find an inverted U-shaped pattern between objective task difficulty and flow. The participants rated flow fairly high in the 0-back task. Due to the signal-detecting nature of the 0-back task, one still has to exert considerable effort and invest attentional resources in order to perform well. Yet, because it was within reach of the all the participants to obtain a good performance on the 0-back task (the mean correct rate was 96.8%), it may have been particularly satisfying and rewarding, thus associated with relatively high levels of flow.

Another point of interpretation relates to the P300. It was argued that the P300 partly reflects the regulatory functions of the LC-NE system in the decision-making process of task engagement^29,31^. This notion mainly builds on the similarity between P300 properties and the properties of the LC-NE system^47^. It received support from early lesion studies^48,49^ as well as recent causal evidence from animal studies^47^. However, unlike the relative robust relation between pupil dilation and the LC-NE system, the relation between P300 and the LC-NE system remains more controversial^50^. In the present study, we indeed found a significant positive within-person correlation between pupil dilation and P300 amplitude, which is in line with the idea that both reflect LC-NE system activity. However, we did not find support for a positive relation between flow and P300 amplitude. To rule out the possible task difference effect on P300 amplitude that may result from the 0-back task, we ran additional analyses on data from which the 0-back task was removed. Again, no significant relationship between flow and P300 amplitude was found (detailed results are provided in the supplementary information p. 2–5). We believe that this may be relevant to the involvement of other mechanisms. Although pupil dilation as well as the P300 have both been linked to the LC-NE system, the literature makes clear that neither may be regulated exclusively by LC-NE system activity^51^. For example, the P300 is also affected by dopaminergic activity^52^. A fMRI study suggested that the ventral striatum also plays a role in regulating P300^53^. Therefore, the involvement of such other mechanisms may serve as a possible explanation of why we found a positive association between pupil dilation and P300 amplitude, but they did not perfectly mirror each other in predicting flow. As this is the first study that examines this relationship (except for indirect evidence from a dual-task^34^), we encourage future studies to further explore this relationship.

Moreover, the task manipulated by task difficulty to induce different levels of flow was not optimal. Specifically, to create enough variance in difficulty, we included a 0-back, 1-back, 2-back, and 3-back task in our experiment. However, the 0-back task is different in nature from the other three tasks, as it does not involve updating memory. Even though we addressed the different target image issue by including only the non-target trials in the analyses, the different cognitive process involved in these tasks is still worth noting. Thus, future studies are encouraged to refine the experimental design to make the conditions more comparable.

All in all, the present study provides initial, but robust empirical indications for a role of the LC-NE system in regulating psychological flow. These findings may yield subsequent research on this topic and contribute to building a neuropsychological model of flow.

## Methods

### Participants

This study was approved by the Research Ethics Review Committee of the Department of Psychology, Education & Child Studies, Erasmus University Rotterdam. Informed consent was obtained from all participants. All the experimental procedures were performed in accordance with the guidelines and regulations approved by the Research Ethics Review Committee. Thirty-seven undergraduate students between the ages of 18–30 (mean age = 20.44 ± 2.64) participated for study credits. All participants are right-handed. One participant was excluded due to noisy signal in both EEG data (more than 2/3 data loss)^54^ and pupillometry data (more than 60% data loss)^55^, resulting in a final sample size of 36 persons (23 female, 13 male). All participants included in the final sample were well-rested and in good health, with normal or corrected-to normal vision. Participants were asked to withhold of caffeine and alcohol during the 24 h before the experiment.

### Material and design

**Figure 5:**
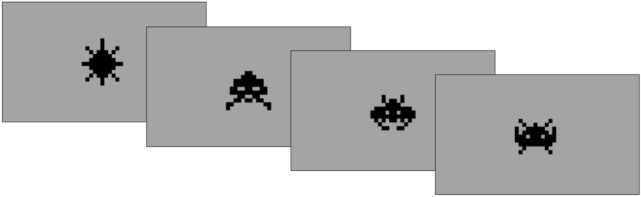
The stimuli in the gamified *n*-back task.

Four gamified versions of the *n*-back task^56^ with different difficulty levels were used. We created the gamified version of the *n*-back task in order to make it more engaging and increase the probability of flow. We reframed the task into an interstellar mission, and the stimuli were described as residents or virus on a planet, rather than simply letters or numbers. In the gamified *n*-back version, four black-colored alien-like figures were used as stimuli (see Fig. 5). Participants were asked to complete *n*-back task sessions comprising 4 assigned difficulty levels, namely, 0-back, 1-back, 2-back, and 3-back, in a random sequence. Participants played the role of a gatekeeper on a spaceship to let residents on board but kick viruses out. In the experimental task, a figure is a virus if it is the same as the one presented *n* figures before, otherwise, it is a resident. Accordingly, they respond on the corresponding key on a keyboard (1 = ‘in’, 2 = ‘out’). Figures are presented randomly with a match (virus) rate of 30% on a 24-inch screen with a resolution of 1920 × 1200 pixels.

### Procedure

Before the experiment, participants were asked to fill out questionnaires regarding their age, gender, handedness, major, and general health information. After calibration of the eye-tracker, participants started with a passive viewing period to get familiar with all the stimuli. The passive viewing consisted of 20 trials. After that, participants were given unlimited opportunity to practice the four tasks. After 20 trials practice for each task, participants chose to have another round of practice or move on to the real test. The main experiment consisted of four blocks in a randomized order (0-back block, 1-back block, 2-back block, and 3-back block). Each block consisted of 80 trials with a target/match rate of 30%. For each trial, a stimulus appeared on the center of the gray screen for 500 ms followed by an interstimulus interval randomized at 3550 ms to 4050 ms. Participants were instructed to respond as correctly and quickly as possible. Responses later than 1500 ms after stimuli onset were considered no response. It took 5–7 min to complete each block. After each block, the participants were asked to indicate their subjective feelings during the task. The total duration of the experiment was 40–50 min.

### Measurements

*Psychological flow* was operationalized by two items from the state flow scale^57^ that capture the core of the flow experience. The items were “I was ‘in the zone’” and “It felt like I was ‘in the flow’ of things”. Participants answered along a 5-point Likert scale from “1 = strongly disagree” to “5 = strongly agree”. Cronbach’s α of the scale measured in each task revealed good reliability (α(*0-back*) = 0.89, α(*1-back*) = 0.86, α(*2-back*) = 0.79, α(*3-back*) = 0.83).

*Task difficulty* was indexed by the objective task difficulty of the levels of n-back task and the subjective task difficulty, which was indexed by the relative difficulty compared with participants’ personal skills. The subjective task difficulty was measured by asking “To what degree did the demands of the task matches your skill in the task?” Participants answered on a scale ranging from “-3 = much lower than my skill” to “0 = match” to “3 = much higher than my skill”. The scale was later transferred into 1–7 for the convenience of statistical analysis and interpretation.

Even though we expected that the 4 assigned difficulty levels would, on average, induce different levels of flow, previous studies suggested that mainly the subjective experience of task difficulty in relation to one’s own skill is relevant for flow to occur^8,39^. In other words, what is important is the perceived balance between personal skill and challenges^41^. Thus, the current study took both objective and subjective task difficulty into account to better understand the nature of flow.

*Continuous EEG* was recorded using a Biosemi ActiveTwo recording system. Sixty-four electrodes used in this study were mounted in an elastic cap using the international 10/20 system sites. A common mode sense (CMS) electrode is located near the site PO1, with a driven right leg (DRL) electrode located near the site PO2. The horizontal electrooculogram (EOG) was recorded from electrodes placed lateral to the external canthi and was used to detect horizontal eye movements; vertical EOG recorded from two electrodes placed above and below the left eye were used to detect eyeblinks and vertical eye movements.

According to the literature, usually, low electrode impedance is important for obtaining high quality data^58^. Considering that active electrodes,-which provide amplifiers close to the origin skin side and impedance transformation-, were used in our recording: the input impedance is very high (so the EEG voltages are not influenced), while the output impedance is very low (< 1 Ohm). Consequently, the interference current flows via low impedances (the output of the active electrode) and cannot generate significant interference voltages anymore. Because the actual electrode impedance is not a relevant variable when active electrodes are used, it is suggested that the level of DC offset should be used as an alternative indicator for the quality of the electrode contact^59^. Following the BioSemi guidelines (http://www.biosemi.com/publications/artikel3.htm), we checked scalp EEG electrode offsets, ensuring < 20 mV was observed at each channel^60^. Online signals were recorded with a sample rate of 512 Hz and 24-bit A/D conversion.

*Pupil diameter* was recorded continuously during the experimental task with a Tobii Pro Fusion. The sampling rate was 250 Hz.

### Data processing and analysis

There are two types of trials in the n-back task, target trials (that match the nth previous trial) and non-target trials (no match with the stimulus nth previous trial). In the 0-back task, we used a unique image as target, while the other 3 images were used repeatedly in 0-back non-target trials and all trials in other tasks. The nature of the tasks makes the 0-back task different from the other tasks, especially the target trials. However, we considered the 0-back a relevant task in the design as it reflects a rather easy task. This is in line with the data, as most participants evaluated that task as “much lower than my ability”. It would be unbalanced, however, to only analyze the target trials of the 1, 2, and 3 back task. According to the distribution of the data without 0 back task, when we regroup the data according to subjective task difficulty, the group “much lower than my ability” results in only one data point. If we exclude all the data from the 0-back task, there is a risk that only part of the effect and pattern is captured. Since we are trying to find a U-pattern, such ceiling effect could result in a wrong conclusion. Accordingly, we decided to run analyses on all four tasks. Moreover, according to a previous study, target trials and non-target trials elicit different physiological features^61^. Therefore, to ensure the data are comparable, we included only the correct non-target trials in four tasks in the statistical analyses. This criterion was used for both EEG data and pupillometry data.

EEG signal processing and analysis were performed in MATLAB using the EEGLAB toolbox^62,63^. The raw data was filtered with a 0.1–30 Hz bandpass. EEG signals were referenced to the computed average. Then, data was segmented for each task condition. Trials were then segmented beginning 500 ms prior to the onset of the stimulus and continuing for 2000 ms post stimulus, baseline correction was performed using the 200 ms prior to stimulus onset. Independent component analysis (ICA) was performed for each participant to identify and remove components that are clearly associated with eyeblinks, and muscle movement as assessed by visual inspection of the waveforms and the scalp distributions of the components^64^. Epochs containing artifacts were removed by multiple methods, including abnormal values exceed ± 75 μV, abnormal trends with a slope greater than 50 μV/epoch and R^2^ more than 0.3, and improbable data exceeding 5SD single-channel limit. P300 was calculated as the mean value between 280 and 380 ms on the Pz electrode^65,66^.

Pupillometry data processing and analysis were performed in MATLAB using the CHAP toolbox^67^. We removed outlier samples with Z-scores larger than 2.5^68^. For each participant, we excluded from the analyses the trials with more than 20% of missing values. Trials with no responses or incorrect responses were deleted. Artifacts and blinks were detected using the algorithm from Hershman et al.^69^ and missing values were filled using linear interpolation. We excluded participants with more than 60% trials identified as outliers in each n-back condition. This criterion was set according to current data quality as well as previous studies^55^. It was suggested by Winn and colleagues that there should be at least 16–18 good recordings of pupil size for each condition for an experiment. The 60% criteria used in our analysis was calculated based on the recommended number of good trials, the overall trials in each block, overall correct rate, and target rate. Least number of valid trials (16–18) < 80(overall trials in each block) × 0.8(average correct rate) × 0.7(non-target rate) × (1 − n%) (percentage of trials to keep). With this approach, we came to the exclusion criteria at 60%. The final sample size used in pupillometry data analysis was 32. We averaged the pupil diameter 500 ms before stimuli as baseline pupil diameter. The peak between 500 and 2000 ms was extracted as peak pupil diameter. Pupil dilation was calculated by subtracting baseline pupil diameter from peak pupil diameter.

Pupil size and EEG were recorded using different devices which resulted in different data quality. There are two possible reasons that we lost more eye-tracking than EEG data. First, compared to EEG measures, pupil recordings are more likely influenced by external factors. Mathot and Vilotihevic stated that ‘data quality can differ substantially between participants, for example due to contact lenses, glasses, eye makeup, or other factors reducing the eye tracker’s ability to record the pupil’. However, such factors do not influence EEG and subjective report data. Second, ideally, pupil size data is recorded with constant gaze position, unaffected by eye blinks, eye movements, or recording artifacts. However, in real word, the quality of pupil size data can never attain this ideal. In our experiment, to make the experimental setting less salient and more flow inducible, we did not introduce a chin rest. This trade-off, however, may have affected the recordings as participants, despite instructions to remain still, had more opportunity to move their heads.

To make full use of available data, for analyses involving only flow and P300 amplitude, all data were included (sample size = 36). For analyses involving pupil data, 4 participants were excluded due to poor quality (sample size = 32). Average trials for EEG data included in the final statistical analyses were 43.76. Average trials for Pupil data included in the final statistical analyses were 45.05.

Preprocessed data were further analyzed using R 4.1.2. Model fittings were done using the lme4 package^38^. By fitting mixed-effect (also known as multilevel/hierarchical) models with random intercepts, we can estimate the real fixed within-subject effects, while avoiding any skewing from the individual difference^70,71^. Considering that all effects we were trying to test in our study are within-person effects, mixed effect models with random intercept, which rule out the effect of individual differences, were conducted all along our analysis^50,72^. Model comparison information was gathered through multiple methods. First, all models were compared using both Akiake’s Information Criterion (AIC)^73^ and Bayesian Information Criterion (BIC)^74^. AIC and BIC provide information about the relative quality of models based on the number of free parameters. Thus, the model with lowest AIC or BIC is deemed providing a better fit for the given set of data. Second, to assist the decision making over competing models, we conducted chi-square difference tests. The Chi-square fit index accesses the fit between the hypothesized model and data from the observed variables. A smaller chi-square index indicates better model fit over competing models. As parallel analyses, in the supplementary material we present the results obtained by a traditional repeated measures analysis of variance.

According to the literature, Cohen’s f^2^ (f^2^ = R^2^/(1 − R^2^)) can be used as an adequate effect size measure for mixed effect models^75,76^. We conducted post-hoc power analyses for all the main results based on their conditional R^2^ (ranging from 0.315 to 0.744, which converts to f^2^ ranging from 0.460 to 3.115). With the current sample sizes (32 for pupil data and 36 for the subjective data and EEG data), the observed power ranges from 0.96 to 1(the ideal power of a study is considered to be > 0.8).

## Supplementary Information

Supplementary Information.

## Data Availability

Pre-registration information of the study could be found on OSF (https://osf.io/qtvew). Pre-processed datasets and the code for main analysis of this study are available in the OSF repository (https://osf.io/4aqry/). Raw datasets are available from the corresponding author upon reasonable request.
