# Supplementary material for: Changes in pupil dilation and P300 amplitude indicate the possible involvement of the locus coeruleus-norepinephrine (LC-NE) system in psychological flow
Source: Sci Rep. 2023 Feb 2;13:1908. doi: 10.1038/s41598-023-28781-z (PMC9894923; doi:10.1038/s41598-023-28781-z)
Supplement: Supplementary file 1 — Supplementary Information. [file 41598_2023_28781_MOESM1_ESM.pdf]

Changes in Pupil Dilation and P300 Amplitude Indicate the Possible Involvement of the Locus Coeruleus-Norepinephrine (LC-NE) System in Psychological Flow

Hairong Lu[1*], Dimitri van der Linden[1], Arnold B. Bakker[1,2]

December 8, 2022

[1] Department of Psychology, Education, and Child studies, Erasmus University Rotterdam, the Netherlands

[2] Department of Industrial Psychology and People Management, University of Johannesburg

Address for correspondence:

Hairong Lu*, Department of Psychology, Education, and Child studies, Erasmus University Rotterdam, 3062 PA, Rotterdam, the Netherlands.

Tel: +31 06 3393 0958; Email: lu@essb.eur.nl

Supplementary Information

Content

1 Results of correct target trials ………………………………………………………. 3

1.1 Pupillometry results of correct target trials ……………………………………. 3

1.2 ERP results of correct target trials ………………………………………..…. 4

1.3 Correlations of pupil dilation and p300 with flow ……………………………... 5

2 ERP results after removing 0-back task ……………………………………….... 7

3 Practice information ………………………………...………………………………. 9

4 Performance results ……………………………...……………………………….. 10

5 Measures of flow ……………………………………………………………………... 11

6 ANOVA results ……………………………………………………………………. 16

7 Tables ……………………………………………………………………………………. 18

1. Results of correct target trials

1.1 Pupillometry results of correct target trials

For correct target trials, the relationship between stimuli-evoked pupil dilation and objective task difficulty fitted the inverted U-shape, see Fig. S1C. The quadratic model fit was significantly better than a linear model (Objective task difficulty: $\beta_{(OTD^2)}$ = -0.029, p < 0.001, B = -0.245, Conditional R^2=0.692, Marginal R^2 = 0.059, $\Delta\chi^2_{(1)}$ = 14.515, p < 0.001). However, the relationship between stimuli-evoked pupil dilation and subjective task difficulty failed to fit the quadratic pattern, see Fig. S1D (Subjective task difficulty: $\beta_{(STD^2)}$ = -0.006, p = 0.080, B = -0.139, Conditional R^2=0.654, Marginal R^2 = 0.023, $\Delta\chi^2_{(1)}$ = 3.099, p = 0.078).

Baseline pupil diameter showed significant positive linear trends with increasing objective and subjective task difficulty (Objective task difficulty: $\beta_{(OTD)}$ = 0.085, p < 0.001, B = 0.225, Conditional R^2=0.874, Marginal R^2 = 0.050; Subjective task difficulty: $\beta_{(STD)}$ = 0.070, p < 0.001, B = 0.248, Conditional R^2=0.863, Marginal R^2 = 0.061), see Fig. S1E and Fig. S1F. Summarized model comparisons are presented in Table S3.

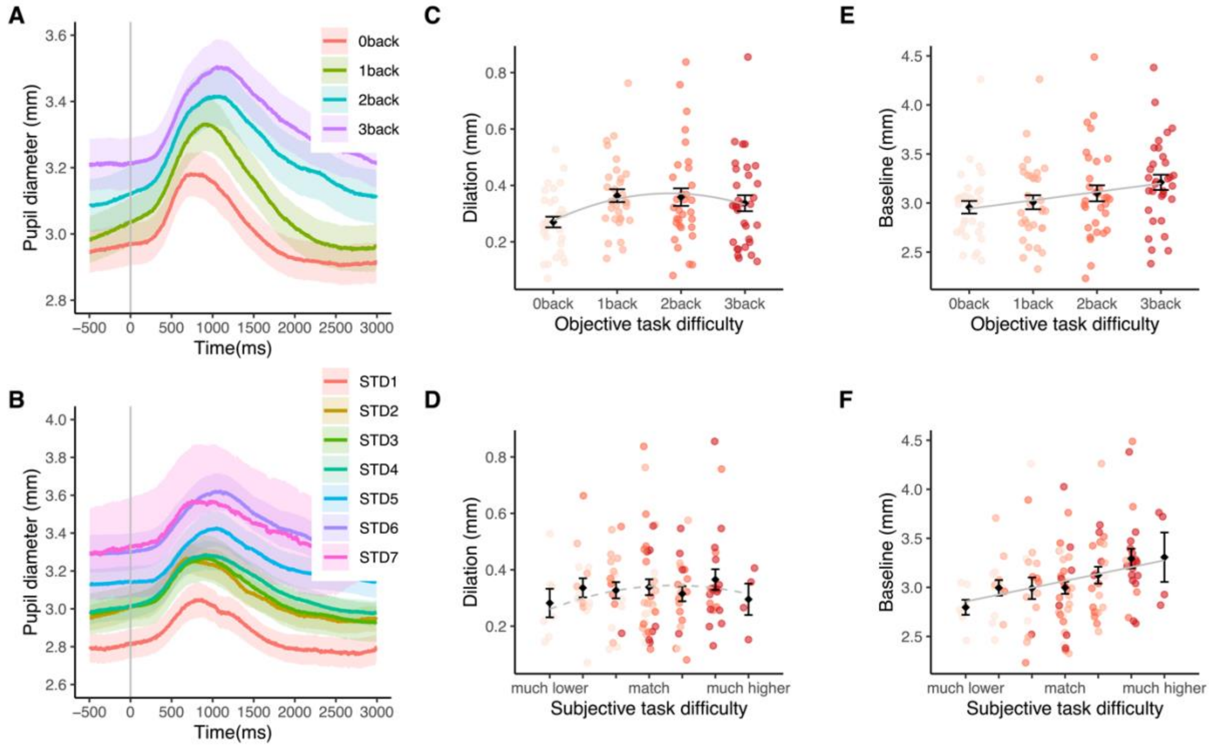

Fig. S1| Pupil diameter changes as function of task difficulty (correct target trials). A) Grand averaged continuous pupil diameter in four n-back tasks. B) Grand averaged continuous pupil diameter in seven subjective task difficulty groups. C) Quadratic relationship between objective task difficulty and stimuli-evoked pupil dilation. D) Quadratic relationship between subjective task difficulty and stimuli-evoked pupil dilation. E) Linear relationship between objective task difficulty and baseline pupil diameter. F) Linear relationship between subjective task difficulty and baseline pupil diameter. The means ± standard errors are shown.

1.2 ERP results of correct target trials

P300 amplitude of the correct target trials failed to fit the inverted U-shapes with objective and subjective task difficulty (Objective task difficulty: $\beta_{(OTD^2)}$ = -0.158, p = 0.292, B = -0.069, Conditional R^2=0.620, Marginal R^2 = 0.034; Subjective task difficulty: $\beta_{(STD^2)}$ = -0.015, p = 0.807, B = -0.012, Conditional R^2=0.614, Marginal R^2 = 0.035), see Fig. S2C and Fig. S2D.

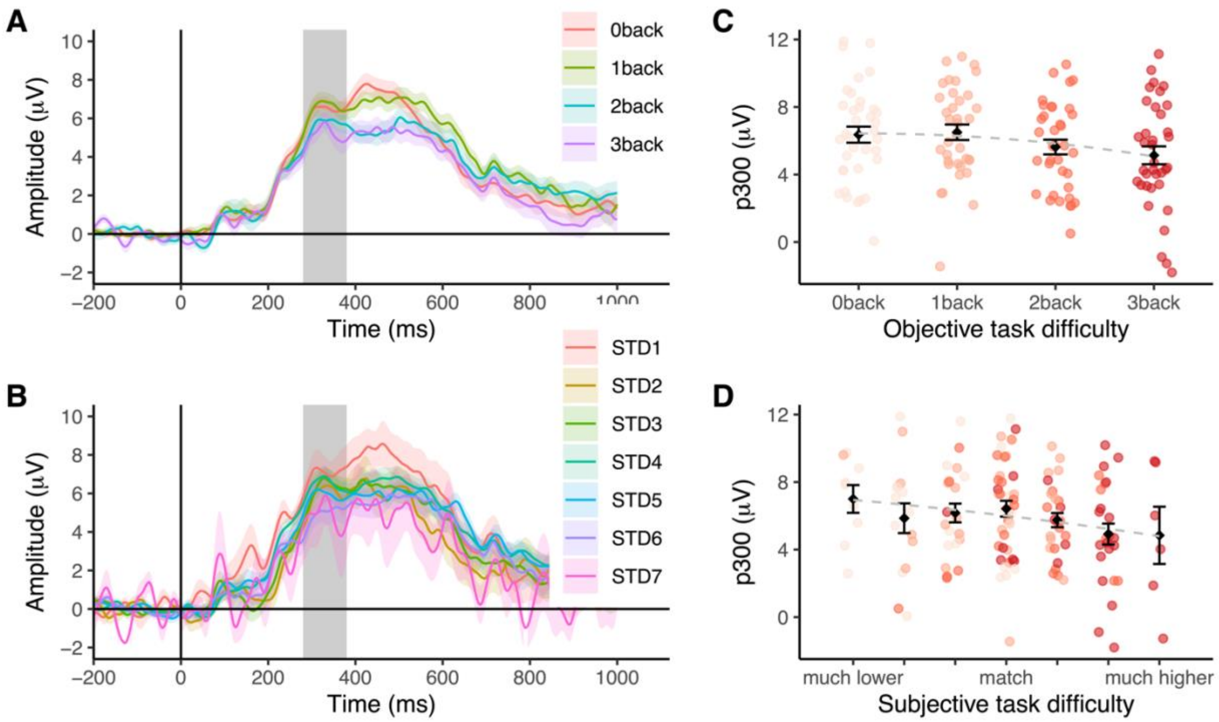

Fig. S2| ERP changes in different task difficulty conditions (correct target trials). A) Grand averaged continuous ERP waveform in four n-back tasks. B) Grand averaged continuous ERP waveform in seven subjective task difficulty groups. The grey shading indicates the time window where we extract P300 value. C) The relationship between objective task difficulty and EEG P300 amplitude failed to fit the quadratic model. D) The relationship between the subjective task difficulty and EEG P300 amplitude failed to fit the quadratic model. The means ± standard errors are shown.

1.3 Correlations of pupil dilation and p300 with flow (correct target trials)

Correlations of pupil dilation and P300 with flow were tested by regressing flow score to these two measures. For correct target trials, the correlation of pupil dilation with flow fit a linear trend (β = 0.020, p <0.05, B = 0.145, Conditional R^2=0.621, Marginal R^2 = 0.021), see Fig. S3A. The correlation of p300 failed to fit a linear trend with flow as well (β = 0.187, p = 0.342, B = 0.068, Conditional R^2=0.571, Marginal R^2 = 0.005), see Fig. S3B.

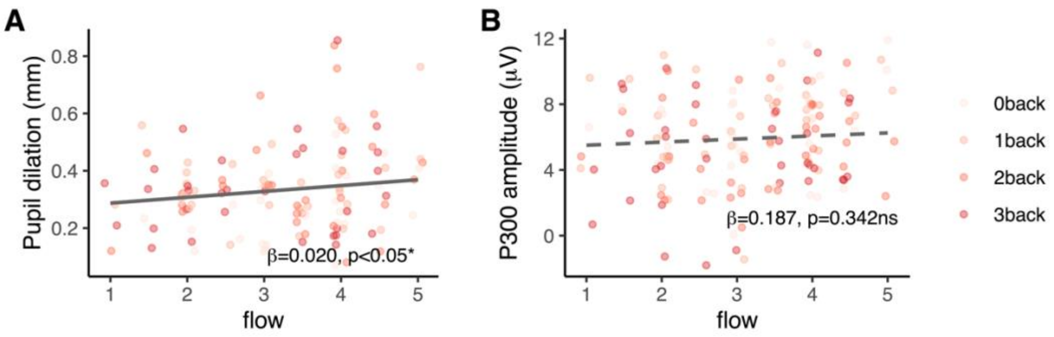

Fig. S3| A) The relationship between pupil dilation and flow failed to fit a linear model B) The relationship between P300 amplitude and flow failed to fit a linear model.

2. ERP results after removing 0-back task

Below, we present the P300 amplitude related results of the data after removing the 0-back task. Note: After removing the 0-back task, only one data point was left in the ‘much lower than my skill’ group’. This confirms that in line with our initial design, the 0-back was considered the easiest task. The 1-back task was already considered relatively challenging.

2.1 P300 amplitude changes across different task difficulties after removing 0-back task

The relationship between P300 and objective task difficulty fit a linear rather than quadratic model (linear model: $\beta_{(OTD)} = -0.665$, $p < 0.001$, B = -0.205, Conditional R^2=0.730, Marginal R^2 = 0.041; quadratic model: $\beta_{(OTD^2)} = 0.003$, p =0.993, B = 0.001, Conditional R^2=0.727, Marginal R^2 = 0.041), see Fig. S4A. The relationship between P300 and subjective task difficulty fit a linear rather than quadratic model (linear model: $\beta_{(STD)} = -0.567$, $p < 0.001$, B = -0.283, Conditional R^2=0.745, Marginal R^2 = 0.077; quadratic model: $\beta_{(STD^2)} = -0.116$, p =0.111, B = -0.077, Conditional R^2=0.746, Marginal R^2 = 0.086), see Fig. S4B.

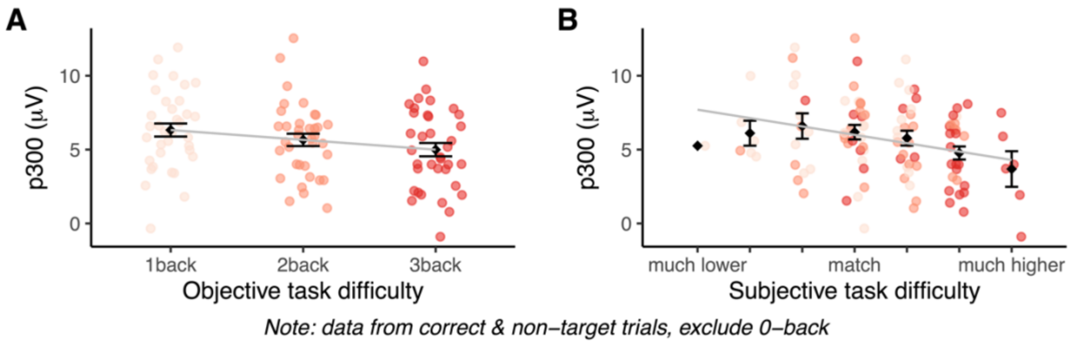

Fig. S4| A) The relationship between objective task difficulty and EEG P300 amplitude (removed 0-back task) fit a negative linear trend. B) The relationship between the subjective task difficulty and EEG P300 amplitude (removed 0-back task) fit a negative linear trend. The means ± standard errors are shown.

2.2 The relationship between p300 and flow after removing 0-back task

Linear relationship between P300 and flow was not significant ($\beta_{(flow)} = 0.200$, $p = 0.344$, $B = 0.013$, Conditional R^2=0.661, Marginal $R^2 = 0.007$), see Fig. S5.

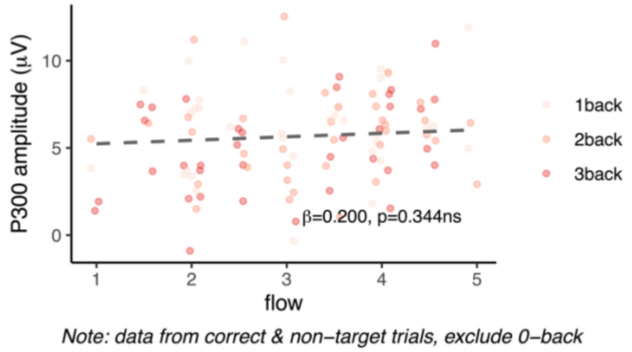

Fig. S5| The relationship between P300 and flow (0-back task removed) failed to fit a linear trend.

3. Practice information

Overall, 13 participants practiced the task for two rounds (one round equals to 20 trials * 4 blocks), 22 participants practiced the task for one round, and two participants requested to skip the practice halfway of their second round of practice (practiced 92 trials and 100 trials separately).

We checked the relationship between the number of practice trials and pupil dilation and p300 amplitude. The results show that there was no relationship between the amount of practice and the physiological measures (pupil dilation ~ amount of practice: $\beta_{(practice\_num)} = 0.0002$, $p = 0.516$, Conditional R^2=0.003, Marginal $R^2 = -0.004$; P300 amplitude ~ amount of practice: $\beta_{(practice\_num)} = -0.003$, $p = 0.621$, Conditional R^2=0.002, Marginal $R^2 = -0.005$.

4. Performance Results

Descriptive information of performance (correct rate, d-prime and reaction time) in objective and subjective task difficulty groups are shown in Fig. S6.

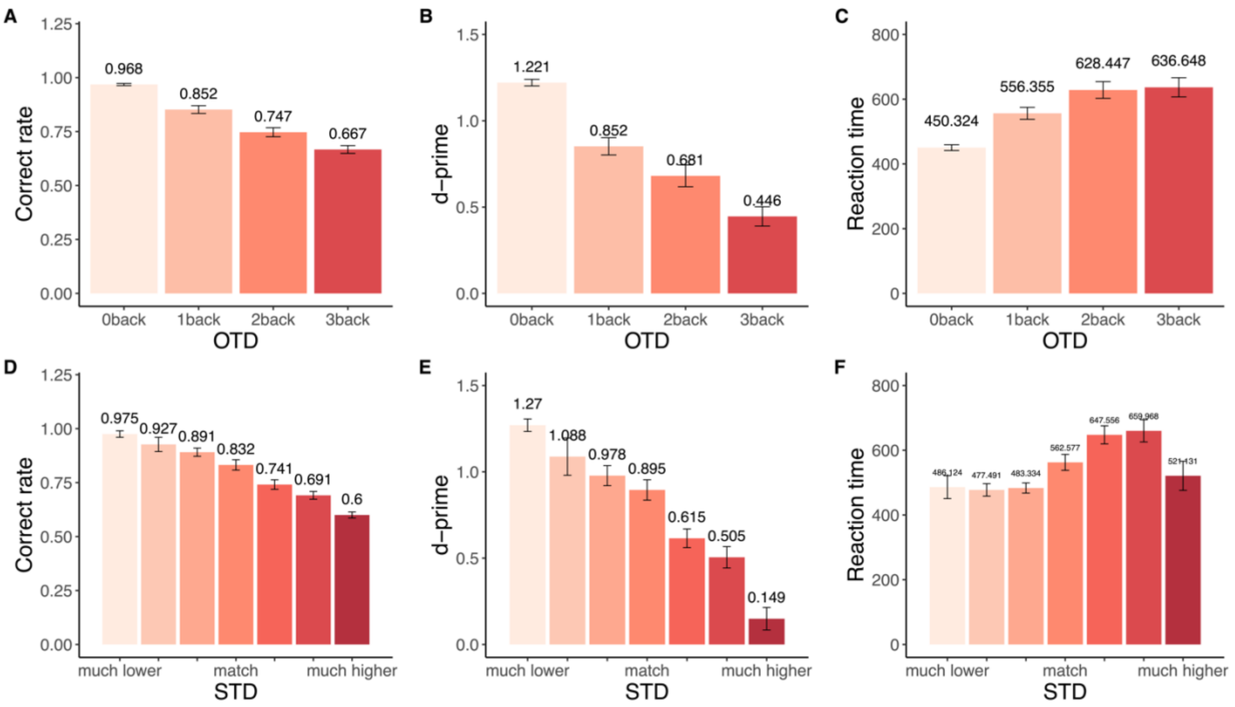

Fig. S6| Descriptive information of performance in different difficulty groups. The means ± standard errors are shown

We tested the relationship between flow and task performance by regressing flow on the d-prime in a mixed effect model and added objective task difficulty as a control variable. The result suggested that there was a positive relationship between flow and task performance ($\beta_{(flow)}$ = 0.105, $p < 0.001$, B = 0.269, $\beta_{(OTD)}$ = -0.240, $p < 0.001$, B = -0.659, Conditional R^2=0.764, Marginal R^2 = 0.527).

5. Measures of flow

In addition to the two general flow items, participants were also asked to report nine psychological aspects that assumed to co-occur with flow -- here we referred to flow characteristics. Items are adapted from the short flow state scale (Jackson, Martin, & Eklund, 2008). They are 1) Competence: “I feel I am competent enough to meet the high demands of the situation”; 2) Automaticity: “I did things spontaneously and automatically without having to think”; 3) Goal clarity: “I had a strong sense of what I wanted to do”; 4) Performance awareness (or feedback): “I had a good idea about how well I was doing while I was involved in the task”; 5) Focus: “I was completely focused on the task at hand”; 6) Control: “I had a feeling of total control over what I was doing”; 7) Little self-referential thinking: “ I was not worried about what others may have been thinking of me”; 8) Distorted time perception: “The way time passed seemed to be different from normal”; 9) Rewarding (or autotelic property): “I found the experience extremely rewarding”. Participants answered along a 5-point Likert scale from “1=strongly disagree” to “5=strongly agree”.

Considering that previous studies, mainly field studies, used the 9-item short flow scale as a whole to measure psychological flow, we checked the Cronbach’s alpha of the 9-item short flow scale and 2-item general flow scale for each task in our experiment. It shows that the Cronbach’s alphas of the 2-item general flow scale were satisfying, however, the 9-item short flow scale rendered poor internal consistencies (all lower than 0.7), see Table S1. Descriptive information of the 11 flow-relevant subjective measures in different task difficulty conditions are shown in Fig. S7.

Table S1| Cronbach's alpha

	Cronbach's alpha	
**Task**	**2-item general flow scale**	**9-item short flow state scale**
0-back task	0.886	0.57
1-back task	0.864	0.461
2-back task	0.787	0.635
3-back task	0.831	0.546

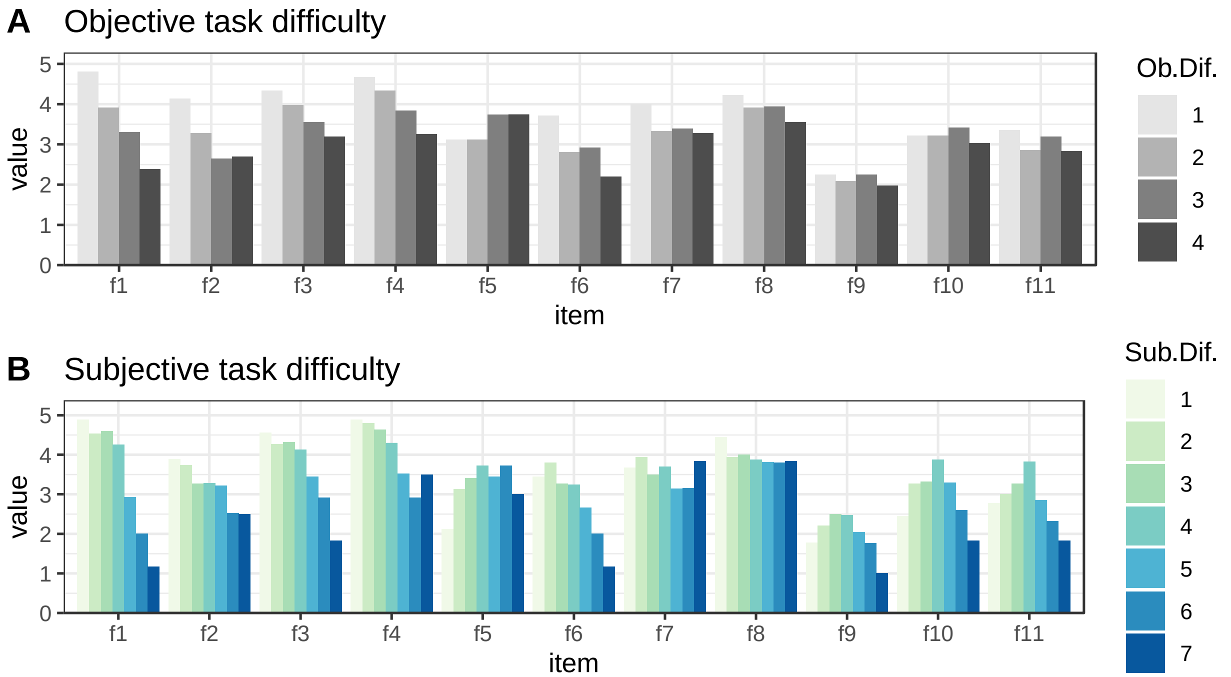

Fig. S7| Mean scores of flow-relevant subjective measures in A) objective task difficulty (0back, 1back, 2back, and 3back), B) subjective task difficulty (1= much lower than my skill, 3 = match, 7 = much higher than my skill)

1. I felt I was competent enough to meet the demands of the situation.

2. I did things spontaneously and automatically without having to think.

3. I had a strong sense of what I wanted to do.

4. I had a good idea about how well I was doing while I was involved in the task.

5. I was completely focused on the task at hand.

6. I had a feeling of total control over what I was doing.

7. I was not worried about what others may have been thinking of me.

8. The way time passed seemed to be different from normal.

9. I found the experience extremely rewarding.

10. I was "in the zone".

11. It feels like I am "in the flow" of things.

We then explored the factor structure of the combined 11 flow-relevant subjective measures by conducting factor analysis with principal components (component extracted based on Eigenvalues greater than 1) and Varimax rotation methods for each task. We find that in the four tasks, the two general flow items always loaded into the first component extracted from all items while loading situations for the other 9 items was inconsistent. See Table S4 for summarized factor loading information.

To further explore the relationship between different flow characteristics and general flow state induced by our experiment, linear mixed models with random intercepts were ran separately. As shown in Fig. S8, a significant positive relationship with the general flow experience was found for competence ($\beta = 0.407$, $p < 0.001$), automaticity ($\beta = 0.216$, $p < 0.05$), goal clarity ($\beta = 0.396$, $p < 0.001$), performance awareness ($\beta = 0.205$, $p < 0.05$), focus ($\beta = 0.524$, $p < 0.001$), control ($\beta = 0.475$, $p < 0.001$), rewarding ($\beta = 0.439$, $p < 0.001$). However, we did not find a significant effect for 'no worry' ($\beta = 0.004$, $p = 0.965$) and time distortion ($\beta = 0.006$, $p = 0.943$).

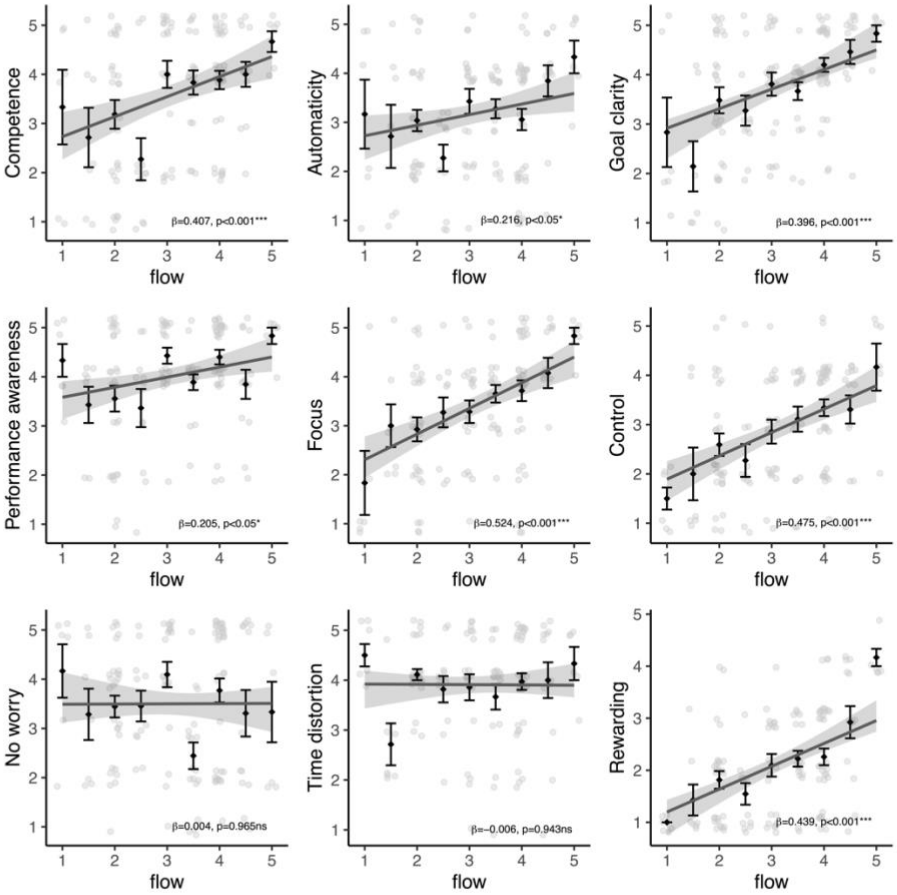

Fig S8| The relationship between different flow characteristics and the general flow experience. The means ± standard errors are shown.

To access which factors contribute to the general flow experience, we then ran a linear mixed model with the 9 assumed characteristics of flow (Model 1: *flow ~ 1 + competence + automaticity + goal clarity + performance awareness + focus + control + no worry + time distortion + rewarding+(1|sub)*). Significant positive effects were found for goal clarity ($\beta = 0.212$, $p < 0.05$), focus ($\beta = 0.301$, $p < 0.001$), and rewarding ($\beta = 0.234$, $p < 0.05$). Then, we ran a linear mixed model with these three significant factors (Model 2: *flow ~ 1+ goal clarity + focus + rewarding+ (1|sub)*). The final regression model ($\beta_{(goal\ clarity)} = 0.294$, $p < 0.001$; $\beta_{(focus)} = 0.265$, $p < 0.001$; $\beta_{(rewarding)} = 0.273$, $p < 0.001$) reached a conditional R^2 of 0.634 and a marginal R^2 of

0.415. It suggests that among all 9 factors, goal clarity, focus, and feeling of reward are positively related to flow experience. Linear mixed model with these three factors account for 63.4% of the total variance of the general flow experience, and the fixed effect of three factors predict 41.5% of the total variance of the general flow experience. The results revealed that even though researchers tend to treat the nine subjective characteristics as indicator of the flow experience, there is a difference in their representativeness. It seems problematic to combine those psychological components to indicate the flow experience.

Prior to the model fitting, predictor multicollinearity was checked by calculating the variance inflation factor (Bruce & Bruce, 2017). For model 1: $VIF_{(competence)} = 3.004$, $VIF_{(automaticity)} = 1.277$, $VIF_{(goal\ clarity)} = 1.889$, $VIF_{(awareness\ of\ performance)} = 1.610$, $VIF_{(focus)} = 1.480$, $VIF_{(control)} = 2.113$, $VIF_{(no\ worry)} = 1.099$, $VIF_{(time\ distortion)} = 1.089$, $VIF_{(rewarding)} = 1.612$. All VIFs were lower than 5, indicating that there is little inflation of the regression coefficient due to collinearity between predictors.

6. ANOVA results

We conducted repeated measures ANOVA analyses for the psychological flow, pupil dilation, and p300 amplitude in four experimental conditions seperately. Further, pairwise t-test with bonferroni-adjusted significance level were conducted to compare differences between each groups. For the comparisons of variables in different subjective task difficulty groups, we reported mean scores with standard error whitout runing statistical analysis. Because different subjective task difficulty groups have different numbers of data points and don't have equal various, which violates the basic assumptions of repeated ANOVA analysis.

First, see Fig. S9A, the results showed that the main effect of n-back task conditions on psychological flow was not significant ($F_{(2.45, 85.83)} = 1.702$, $p = 0.182$, $\eta^2_G = 0.023$) and there was no significant difference of psychological flow between each pair of four n-back task conditions.

Second, see Fig. S9B, repeated measures ANOVA results suggest a significant main effect of n-back task conditions on pupil dilation ($F_{(3, 90)} = 6.77$, $p < 0.001$, $\eta^2_G = 0.038$). Pupil dilation in the 0-back condition was significantly smaller than in the 1-back condition ($t_{(31)} = -4.10$, $p = 0.002$, *Cohen's d* = 0.612) and 2-back condition ($t_{(31)} = -3.71$, $p = 0.005$, *Cohen's d* = 0.602). However, the other 3 conditions didn't show significant differences between each other.

Third, see Fig. S9C, repeated measures ANOVA results suggest a significant main effect of n-back task conditions on P300 amplitude ($F_{(3, 105)} = 10.468$, $p < 0.001$, $\eta^2_G = 0.049$). Pairwise t-test shown that the P300 amplitude in the 0-back condition was significantly lower than the 1-back ($t_{(35)} = -6.14$, $p < 0.001$, *Cohen's d* = 0.623) and partially significantly lower than the 2-back condition ($t_{(35)} = -2.76$, $p = 0.054$, *Cohen's d* = 0.324). Moreover, the P300 amplitude in the 1-back condition was somewhat higher than the 2-back condition – although not significant ($t_{(35)}$ =

2.69, $p = 0.066$, *Cohen's d* = 0.265), and significantly higher than the 3-back condition ($t_{(35)} = 3.85$, $p = 0.003$, *Cohen's d* = 0.460).

Descriptive information about mean score of flow, pupil dilation, and P300 amplitude in different subjective task difficulty groups are shown in Fig S9 D-F.

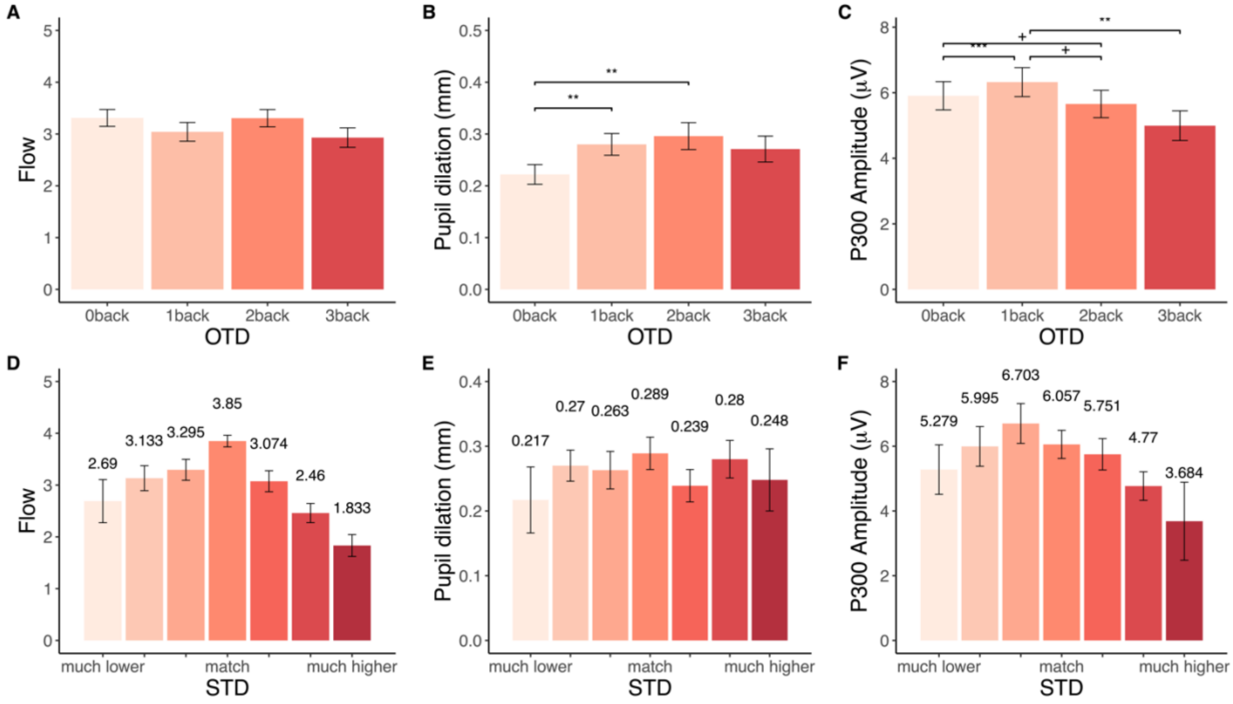

Fig. S9| ANOVA results. A) Psychological flow in four n-back task conditions show no significant difference. B) Comparisons of pupil dilation in four n-back conditions. C) Comparisons of p300 amplitude in four n-back conditions. D) Mean score of flow in different subjective task difficulty groups. E) Mean score of pupil dilation in different subjective task difficulty groups. F) Mean score of P300 amplitude in different subjective task difficulty groups. The means ± standard errors are shown.

Table S2| Model comparisons (correct nontarget trials)

Type	Models	Items	Estimate	Std.Error	df	t value	*Pr(>|t|)*	*Standard Est.*	*Conditional R^2*	*Marginal R^2*	AIC	BIC	chisq	df	*pr(>Chisq)*
		(Intercept)	3.367	0.203	129.863	16.622	<0.001***	0.000							
Linear model	flow ~ 1 + OTD + (1|sub)	OTD	-0.088	0.064	107.000	-1.375	0.172	-0.094	0.344	0.009	410.280	422.160			
		(Intercept)	3.235	0.412	124.000	7.851	<0.001***	0.031							
Quadratic		OTD	0.044	0.364	106.000	0.120	0.905	-0.094							
model	flow ~ 1 + OTD + $(OTD)^2$ + (1|sub)	(OTD)2	-0.026	0.072	106.000	-0.367	0.714	0.032	0.341	0.009	412.150	427.000	0.137	1	0.711
		(Intercept)	3.501	0.243	135.947	14.388	<0.001***	0.000							
Linear model	flow ~ 1 + STD + (1|sub)	STD	-0.086	0.052	130.713	-1.669	0.098	-0.127	0.315	0.160	409.430	421.310			
		**(Intercept)**	**1.609**	**0.402**	**133.952**	**4.001**	**<0.001*****	**0.328**							
**Quadratic**		**STD**	**1.055**	**0.207**	**121.875**	**5.085**	**<0.001*****	**-0.204**							
**model**	**flow ~ 1 + STD + $(STD)^2$ + (1|sub)**	**(STD)2**	**-0.145**	**0.026**	**121.979**	**-5.664**	**<0.001*****	**-0.330**	**0.401**	**0.171**	**381.950**	**396.800**	**29.482**	**1**	**<0.001*****
		(Intercept)	0.226	0.025	64.988	9.173	<0.001***	0.000							
Linear model	dilation ~ 1+ OTD + (1|sub)	OTD	0.016	0.006	95.000	2.879	0.005**	0.142	0.695	0.020	-230.090	-218.690			
		**(Intercept)**	**0.123**	**0.039**	**124.751**	**3.170**	**0.019****	**0.198**							
**Quadratic**		**OTD**	**0.119**	**0.031**	**94.000**	**3.876**	**<0.001*****	**0.142**							
**model**	**dilation ~ 1+ OTD + $(OTD)^2$ + (1|sub)**	**(OTD)2**	**-0.021**	**0.006**	**94.000**	**-3.395**	**0.001****	**-0.200**	**0.726**	**0.045**	**-239.200**	**-224.940**	**11.103**	**1**	**<0.001*****
		(Intercept)	0.234	0.029	90.160	8.180	<0.001***	0.000							
Linear model	dilation ~ 1+ STD + (1|sub)	STD	0.008	0.005	101.900	1.622	0.108	0.094	0.683	0.009	-224.660	-213.250			
		**(Intercept)**	**0.131**	**0.044**	**124.815**	**2.967**	**0.004****	**0.138**							
**Quadratic**		**STD**	**0.070**	**0.021**	**97.248**	**3.330**	**0.001****	**0.067**							
**model**	**dilation ~ 1+ STD + $(STD)^2$ + (1|sub)**	**(STD)2**	**-0.008**	**0.003**	**97.361**	**-3.019**	**0.003****	**-0.139**	**0.719**	**0.032**	**-231.420**	**-217.160**	**8.758**	**1**	**0.003****
		(Intercept)	6.571	0.467	67.972	14.067	<0.001***	0.000							
Linear model	p300 ~1 + OTD + (1|sub)	OTD	-0.340	0.103	107.000	-3.304	0.001***	0.145	0.729	0.021	595.560	607.440			
		**(Intercept)**	**5.221**	**0.730**	**139.977**	**7.153**	**<0.001*****	**0.128**							
**Quadratic**		**OTD**	**1.010**	**0.572**	**106.000**	**1.764**	**0.081**	**-0.145**							
**model**	**p300 ~ 1 + OTD + $(OTD)^2$ + (1|sub)**	**(OTD)2**	**-0.270**	**0.113**	**106.000**	**-2.396**	**0.018***	**-0.129**	**0.741**	**0.031**	**591.860**	**606.710**	**5.698**	**1**	**<0.05***
		(Intercept)	6.850	0.527	93.803	13.010	<0.001***	0.000							
Linear model	p300 ~ 1 + STD + (1 | sub)	balance	-0.275	0.087	114.115	-3.160	0.002**	0.161	0.725	0.026	596.370	608.250			
		**(Intercept)**	**4.877**	**0.778**	**140.939**	**6.271**	**<0.001*****	**0.136**							
**Quadratic**		**STD**	**0.911**	**0.361**	**110.157**	**2.523**	**0.013***	**-0.192**							
**model**	**p300 ~ 1 + STD + $(STD)^2$ + (1 | sub)**	**(STD)2**	**-0.151**	**0.045**	**110.185**	**-3.374**	**<0.001*****	**-0.137**	**0.744**	**0.051**	**587.270**	**602.110**	**11.103**	**1**	**<0.001*****

Note: Correct non-target trials

OTD = objective task difficulty (n-back type); STD = subjective task difficulty

*'***' 0.001 '**' 0.01 '*' 0.05 ';*

Table S3| Model comparisons (correct target trials)

Type	Models	Items	Estimate	Std.Error	df	t value	*Pr(>|t|)*	*Standard Es*	*Conditional*	*Marginal R^2*	AIC	BIC	chisq	df	*pr(>Chisq)*
Linear		(Intercept)	0.284	0.028	72.598	9.969	<0.001***	0.000							
model	dilation ~ 1+ OTD + (1|sub)	OTD	0.019	0.007	95.000	2.761	0.007**	0.147	0.644	0.021	-182.790	-171.380			
		**(Intercept)**	**0.140**	**0.046**	**124.885**	**3.031**	**0.003****	**0.243**							
**Quadratic**		**OTD**	**0.164**	**0.037**	**94.000**	**4.377**	**<0.001*****	**0.147**							
**model**	**dilation ~ 1+ OTD + (OTD)^2 + (1|sub)**	**(OTD)2**	**-0.029**	**0.007**	**94.000**	**-3.917**	**<0.001*****	**-0.245**	**0.692**	**0.059**	**-195.300**	**-181.040**	**14.515**	**1**	**<0.001*****
Linear		(Intercept)	0.284	0.033	97.520	8.507	<0.001***	0.000							
model	dilation ~ 1+ STD + (1|sub)	STD	0.012	0.006	103.200	1.938	0.055.	0.120	0.638	0.014	-179.060	-167.650			
		(Intercept)	0.209	0.054	123.244	3.844	<0.001***	0.138							
Quadratic		STD	0.057	0.026	98.213	2.169	0.33*	0.067							
model	dilation ~ 1+ STD + (STD)^2 + (1|sub)	(STD)2	-0.006	0.003	98.356	-1.767	0.080.	-0.139	0.654	0.023	-180.160	-165.900	3.099	1	0.078.
Linear		(Intercept)	7.044	0.522	87.113	13.498	<0.001***	0.000							
model	p300 ~1 + OTD + (1|sub)	OTD	-0.454	0.134	107.000	-3.393	<0.001***	0.177	0.618	0.031	654.150	666.030			
		(Intercept)	6.252	0.912	138.707	6.856	<0.001***	0.069							
Quadratic		OTD	0.337	0.760	106.000	0.444	0.658	-0.177							
model	p300 ~ 1 + OTD + (OTD)^2 + (1|sub)	(OTD)2	-0.158	0.150	106.000	-0.106	0.292	-0.069	0.620	0.034	655.020	669.860	1.135	1	0.287
Linear		(Intercept)	7.363	0.610	113.843	12.070	<0.001***	0.000							
model	p300 ~ 1 + STD + (1 | sub)	STD	-0.354	0.112	117.917	-3.162	<0.01**	0.190	0.617	0.035	655.540	667.420			
		(Intercept)	7.169	1.000	138.485	7.168	<0.001***	0.012							
Quadratic		STD	-0.238	0.489	112.863	-0.486	0.628	-0.192							
model	p300 ~ 1 + STD + (RTD)^2 + (1 | sub)	(STD)2	-0.015	0.060	112.909	-0.244	0.807	-0.012	0.614	0.035	657.480	672.330	0.062	1	0.804

Note: Correct target trials

OTD = objective task difficulty (n-back type); STD = subjective task difficulty

*'***' 0.001 '**' 0.01 '*' 0.05 ';*

Table S4| Factor loadings of flow items

	0 back task				1-back task				2-back task			3-back task			
Items	Component 1	Component 2	Component 3	Component 4	Component 1	Component 2	Component 3	Component 4	Component 1	Component 2	Component 3	Component 1	Component 2	Component 3	Component 4
f1	-0.128	**0.673**	0.456	-0.253	0.239	-0.227	0.725	-0.262	**0.503**	**0.504**	-0.005	**0.713**	-0.177	0.316	0.371
f2	-0.357	**0.534**	-0.013	0.209	**0.727**	-0.212	0.038	0.494	-0.092	0.101	**0.935**	0.180	**0.684**	0.346	-0.186
f3	0.015	0.463	**0.503**	0.447	0.000	0.351	**0.725**	0.280	**0.608**	**0.651**	0.091	**0.893**	-0.053	0.141	0.023
f4	0.142	0.470	**0.734**	0.117	0.039	0.101	**0.824**	-0.019	0.151	**0.658**	-0.036	0.037	0.008	-0.112	**0.924**
f5	**0.836**	0.147	-0.190	-0.186	0.153	**0.723**	-0.010	-0.184	**0.784**	-0.220	-0.092	**0.676**	-0.162	-0.158	-0.298
f6	0.353	**0.754**	0.075	0.069	0.407	**0.578**	0.262	-0.093	**0.760**	0.274	-0.102	**0.547**	-0.327	**0.523**	0.314
f7	-0.049	-0.049	**0.843**	-0.099	-0.149	**-0.636**	0.302	-0.318	-0.367	0.362	-0.376	0.111	**0.731**	-0.201	0.279
f8	-0.049	0.035	-0.052	**0.945**	-0.055	-0.042	-0.009	0.889	0.001	**0.707**	0.121	-0.001	-0.039	**-0.873**	0.129
f9	**0.802**	0.313	-0.105	0.104	0.040	**0.623**	0.347	0.007	**0.828**	0.107	-0.001	0.439	-0.696	0.068	0.125
f10	**0.825**	-0.283	0.120	0.112	**0.787**	0.320	0.006	-0.098	**0.665**	0.271	0.393	**0.856**	0.093	-0.092	-0.002
f11	**0.890**	-0.087	0.172	-0.081	**0.870**	0.285	0.170	-0.116	**0.701**	0.339	0.203	**0.814**	0.250	0.190	0.191
